# The Malaria in Pregnancy Library: a bibliometric review

**DOI:** 10.1186/1475-2875-11-362

**Published:** 2012-10-30

**Authors:** Anna M van Eijk, Jenny Hill, Sue Povall, Alison Reynolds, Helen Wong, Feiko O Ter Kuile

**Affiliations:** 1 Child and Reproductive Health Group, Liverpool School of Tropical Medicine, Pembroke Place, Liverpool, L3 5QA, UK; 2 Division of Public Health, University of Liverpool, Whelan Building Quadrangle, Liverpool, L69 3GB, UK

**Keywords:** Malaria, Pregnancy, Bibliometrics

## Abstract

**Background:**

The Malaria in Pregnancy (MiP) Library is a bibliographic database that was created by the MiP Consortium in 2005 and is updated every four months using a standardized search protocol. A bibliometric review was conducted of the contents of the Library to determine dynamics in the type, content and volume of literature on malaria in pregnancy over time.

**Methods:**

Data on year of publication, type, language, country of first-author affiliation and content (topic) were extracted from entries in the MiP Library and plotted over time.

**Results:**

By January 2012, the MiP Library contained 5,346 entries, consisting of 3,721 journal articles (69.6%), 697 reports (13.0%), 219 academic theses (4.1%), 92 books or book chapters (1.7%), 487 conference proceedings (9.1%), 68 registered studies (1.3%) and 62 ‘other’ (1.2%). Most of the sources were in English language (87.3%), followed by French (7.5%) and Spanish (1.5%). Over 40% of source material was publicly available online (42.4%) and the remaining with restricted access (35.0%) or otherwise unavailable (22.7%). The number of journal articles related to malaria in pregnancy increased from 41 in the 1960s, to 708 in the 1990s, and 1,895 between 2000 and 2009, and the variety of themes has increased over time. English-language articles were sourced from 737 different journals. The top three journals were the *American Journal of Tropical Medicine and Hygiene* (184), *Malaria Journal* (158) and the *Transactions of the Royal Society of Tropical Medicine and Hygiene* (131).

**Conclusion:**

The last decade has seen a dramatic increase in publications related to malaria in pregnancy, and an increasing proportion of these are publically available online. The MiP Library is a useful, scholarly source for literature and systematic reviews related to malaria in pregnancy.

## Background

Malaria is currently endemic in 99 countries worldwide [1]. In 2010, the number of cases globally was estimated at 216 million, representing a 17% decrease compared to estimates in 2000 [1]. According to recent models, global malaria mortality peaked in 2004 with 1.8 million deaths, and decreased thereafter to 1.2 million deaths in 2010 [2]. This decline is primarily attributed to the increase in funding and expansion of control efforts seen in the last decade [1,3]. Pregnant women comprise one of two groups particularly vulnerable to malaria, the other being children. Malaria during pregnancy can have devastating consequences to both mother and developing foetus and is associated with mild to severe maternal illness, maternal anaemia, spontaneous miscarriage, stillbirth, preterm delivery and foetal growth retardation [4]. Malaria is the most important preventable cause of low birth weight in malaria-endemic areas in sub-Saharan Africa, which in turn is associated with increased susceptibility to illness and death in early life [4,5]. WHO recommends a package of intermittent preventive treatment (IPT) with sulphadoxine–pyrimethamine (SP) and use of insecticide-treated bed nets (ITNs) for the prevention of malaria combined with effective case management of clinical malaria and anaemia in sub-Saharan Africa [6]. There are no standardized prevention policies for malaria in pregnancy in other endemic malaria regions of the world where the focus is on case detection and treatment.

The Malaria in Pregnancy (MiP) Library was created in 2005 by the MiP Consortium in order to assemble all available published and unpublished literature on malaria in pregnancy, and make its database accessible to researchers and other interested members of the public [7]. A bibliometric review of the contents of the MiP Library was undertaken to explore how research interests in malaria in pregnancy have evolved over time; specifically, how the focus of research on malaria in pregnancy has changed, and how increases in malaria in pregnancy publications compare to publications on malaria in children during the same period.

## Methods

The MiP Library is updated every four months through a search of available online literature for new information on malaria in pregnancy using a standardized search protocol (see Table 1 and Additional file 1). In addition, reference lists in the retrieved literature are checked for secondary citations. The published literature includes articles, books, reports, academic theses, and policy guidelines. The unpublished literature includes registered studies, unpublished theses or research, and meeting reports. Whilst there is no language restriction, the focus to date has been on the European family of languages and predominantly English.

**Table 1:** Search strategy and inclusion criteria Malaria in Pregnancy Library

	
**PubMed**	("Malaria"[MeSH] OR malaria OR "plasmodium"[MeSH] OR plasmodium OR falciparum OR vivax) AND ("pregnancy"[MeSH] OR pregnancy OR pregnant OR "pregnancy complications"[MeSH] OR "placenta"[MeSH] OR placenta OR placental OR "fetus"[MeSH] OR fetus OR fetal OR foetus OR foetal OR gravidity OR primigravid* OR "low birth weight" OR LBW OR antenatal OR congenital)
**Generic**	(malaria OR plasmodium OR falciparum OR vivax OR malariae) AND (pregnancy OR pregnant OR “pregnancy complications” OR placenta* OR fetus OR fetal OR foetus OR foetal OR gravid* OR “low birth weight” OR LBW OR antenatal OR congenital)
**Minimum**	Malaria AND Pregnan*

General information for each relevant entry is recorded, such as author, date of publication, and title. Entries are categorized according to reference type, content and topic. The reference types include journal article, report, thesis, book or book chapter, conference proceeding (abstract), registered study, and other (e.g. electronic citation, presentations, unpublished work). The content type includes: primary research articles (henceforth referred to as original research), review (secondary analysis or discussion of other researchers' primary research), commentary (editorial or letter commenting on others’ primary research), policy document, practice guidelines, programme evaluation, conference summary or report, and other content (e.g. grant proposals or study protocols). Categories are not mutually exclusive such that single entries can contribute to multiple categories. Items which are both available in English and another language are categorized as English. Entries obtained from clinical trial registries are removed from the library once publications of data from the registered studies are entered in the database, to avoid double entries of included studies. A similar procedure is maintained for conference proceedings (abstracts). Institute, location and country of the first author’s affiliation (e.g. for journal articles) were recorded or the institute or agency which published the material (e.g. for reports). Information was collected on impact factor (one of the leading proxies for evaluating quality of a journal) [8], the ‘Eigenfactor’ (a rating of the overall importance of a journal), and article influence score (a score which takes into account differences in the citation patterns between areas of research) [9], for the journals in the Library for 2010, which was the latest year for which the factors were available at the time this information was collected [10] and assessed if the journals were still active using the National Library of Medicine Catalogue [11] or individual journal website pages. It was checked if the journal article was indexed in PubMed.

## Analysis

The Library contents was analysed from its earliest entry to January 2012. Malaria in pregnancy was considered the main theme if the title contained any reference to both pregnancy and malaria (Table 2), or if after reviewing the abstract, or main text if no abstract was available, malaria in pregnancy was found to be the main theme. The entries were then categorized (using the abstract or full content analysis if the abstract was not available) according to the following sub-topics of malaria in pregnancy (not mutually exclusive): epidemiology and burden; pathogenesis and immunology; pharmacovigilance and safety; pharmacokinetics; case management; prevention; travel and migration; policy and health systems; social science and anthropology; economics; and diagnosis. Examples of “case management” are drug-efficacy studies and clinical case reports involving patient management, which includes case descriptions of the treatment of malaria in pregnancy or congenital malaria. “Travel and migration” includes articles whereby the population involves either persons from a non malaria-endemic country visiting an endemic country, or persons from a malaria-endemic country migrating to a non-endemic country. “Policy and health systems” includes articles with information on research involved in implementation of preventive strategies, or monitoring and evaluation of programmes to control malaria in pregnancy, e.g. coverage of ITNs or IPTp. Examples of the category “social science and anthropology” includes articles with a focus on perceptions and acceptability of preventive interventions, and knowledge, attitudes and practice regarding malaria from pregnant women and health system perspectives. “Economics” includes studies on cost effectiveness of interventions, economic costs and economic burden of malaria in pregnancy. “Diagnosis” includes articles that involve the use of malaria tests to improve the detection of malaria in pregnancy. The specific analyses were restricted to English language, and assessed the top 10 journals publishing on MiP, and the top 10 publishing countries as defined by the country of the first author affiliation, and calculated the median impact factor and article influence score by country. The current top 15 papers with malaria as main theme by citations were assessed as provided by Google Scholar and “Publish or Perish” using the key words in Table 2[12,13].

**Table 2:** Malaria in pregnancy key words among 2,839 library items with the key words in the title*

**Indicator of pregnancy**	**n**	**%**	**Indicator of malaria**	**n**	**%**
Pregnant* (Trimester, gestation, HCG, risk group or vulnerable group with reference to pregnancy)	1,824	64.2	**Malaria**	2,241	78.9
**Placenta**	505	17.8	***P***. ***falciparum***	555	19.5
**Congenital**	220	7.7	***P***. ***vivax***	42	1.5
**Birth** (birth weight, birth outcome, childbirth, stillbirth, abortion, delivery, parturition, premature, preterm, newborn, neonatal, child-bearing)	195	6.9	***Other species***	47	1.7
**Maternal**	183	6.4	**Anti**-**malarial** (either word or anti-malarial drug)	451	15.9
**IPTp**	162	5.7	**ITN** or net	82	2.9
**Foetus**/**foetal** (Embryo, developmental toxicity, offspring, reproductive toxicity, teratogenic, in utero, trophoblast)	149	5.2	**Insecticide**	46	1.6
**Antenatal**/**prenatal**/**perinatal**/**postpartum** (puerperal, postnatal)	120	4.2	**Other** (e.g. *Anopheles gambiae*, VAR2CSA, haemozoin, malaria specific antibodies)	15	0.5
**Cord**	56	2.0			
**Gravid** (primigravidae, multigravidae)	47	1.7			
**CSA** only	24	0.8			
**Breast milk**	20	0.7			
**Diseases characteristic for pregnancy**, e.g. pre-eclampsia, HELLP syndrome	5	0.2			

Abbreviations: *CSA* chondroitin sulphate A; *HCG* human chorionic gonadotropin (a hormone produced during pregnancy); *IPTp* intermittent preventive treatment in pregnancy; *ITN* insecticide treated nets.

*An item can have more than one key word related to pregnancy or malaria in the title.

Two sub-analyses were conducted to assess time to publication and publication bias. Using the MiP Library archive of registered studies, the number, status and outcome of the registered studies were evaluated that had MiP key words in their title. All sources for registered studies were used (Additional file 1) except for RePorter (previously known as Crisp) because this register provides insufficient detail with regard to study design and progress. The status of registered studies was assessed as: recruiting; not yet recruiting; completed; suspended; terminated, and the outcome of completed studies as published or not yet published. The time interval between the end of completed studies and publication date was also assessed. Abstracts with malaria and pregnancy, or equivalent terms, in their title were evaluated from the last three Multilateral Initiative on Malaria (MIM) conferences held in 2003, 2005, and 2009, the largest conference dedicated exclusively to malaria. An assessment was made whether these abstracts had resulted in a peer-reviewed journal publication by searching for publications (as first, or co-author) of the first author of the abstract and, if yes, the time interval between the conference and publication date was calculated. To compare the dynamics over time of the number of publications related to malaria in pregnancy with that in children, two comparable searches were conducted in PubMed (no language restriction); the Mesh terms for PubMed were used as described in Table 1 to identify articles related to malaria in pregnancy, and the same Mesh terms for malaria in combination with the terms ("Child"[MeSH] OR child OR "Infant"[MeSH] or infant OR newborn OR baby) were used to find articles related to malaria in children. The results were plotted over time.

All analyses were conducted in SAS version 9.2; chi-square tests were used to compare proportions and non-parametric tests to compare median impact factors and article influence scores. A p-value of < 0.05 was regarded significant.

## Results

**Figure 1:**
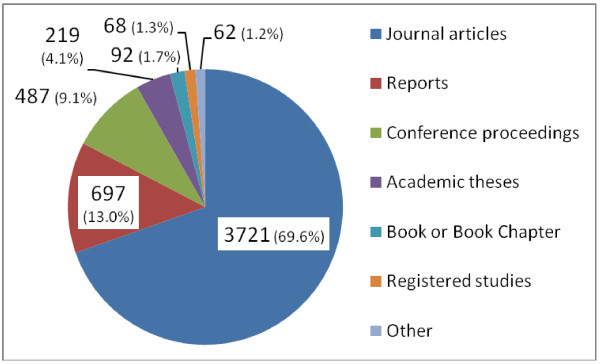
Content of the Malaria in Pregnancy Library by format, January 2012.

**Figure 2:**
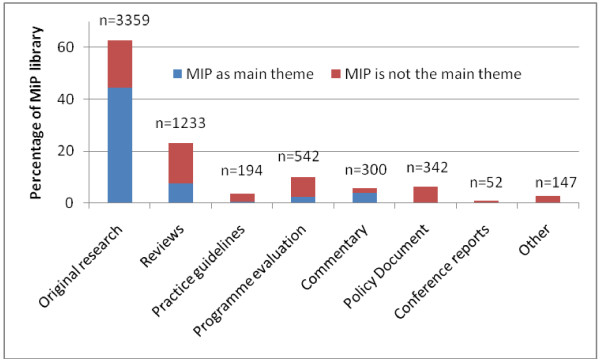
Items by content in the Malaria in Pregnancy Library, January 2012.

**Figure 3:**
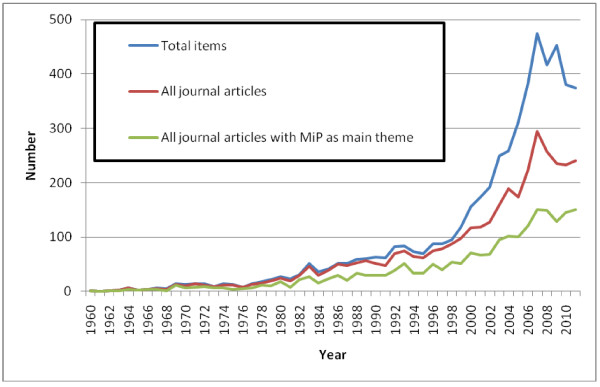
Number of items of Malaria in Pregnancy Library by year since 1960, January 2012.

By January 2012, there were 5,346 entries in the MiP Library, the earliest dating back to 1850. The majority of entries comprised of journal articles (69.6%, Figure 1), and reports and abstracts contributing an additional 13.0% and 9.1%, respectively. In total, 21 languages were represented: English (87.3%) was the most common, followed by French (7.5%) and Spanish (1.5%). Forty two percent of entries were freely available online; for a further 35.0% web access was restricted (registration required), and the remaining 22.7% were not available online. Malaria in pregnancy was considered the main theme for 55.7% of the entries; for 53.1%, malaria and pregnancy key words were in the title (Table 2). Among original research articles, 70.6% had MiP as the main theme; for reviews and programme evaluation this was 33.4% and 23.4% respectively (Figure 2). Before 1960, 74 items with information on malaria in pregnancy were identified; 70 were articles (94.6%) and for 64 of them (91.4%) malaria in pregnancy was the main theme. From 1960 onwards, there was a gradual increase in number of entries, which became exponential by the year 2000 (Figure 3) and reached a peak in 2007 with 474 articles, after which it declined. The same trend was seen for journal articles overall whereas annual number of articles with MiP as main theme seemed to plateau from 2007 onwards (Figure 3). From this point onwards, all analyses are restricted to the 4,668 English language items.

## Journal articles

**Figure 4:**
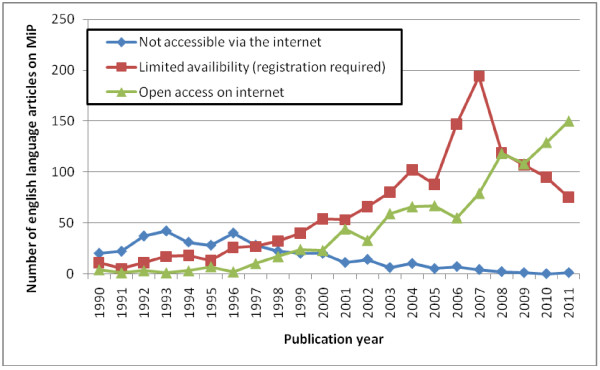
Accessibility of English-language articles through the internet, January 2012.

Journal articles constituted the main publication type in the MiP Library, with 3,194 entries (68.4% of library entries) published in 737 different journals; 624 journals (84.4%) were still active and for 15 journals this information could not be retrieved. An impact factor and article influence score for 2010 was available for 374 (50.8%) and 346 (47.0%) journals respectively. The median (range) was 2.52 (0.15-53.49) and 0.87 (0.01-21.35) respectively, with the highest for the *New England Journal of Medicine* (53.49) and the lowest impact factor for the *Indian Journal of Animal Science* (0.15). The country of publication could be retrieved for 723 journals (97.8%); journals from the USA and the UK were most prevalent (28.6% and 26.7%, respectively), followed by India (5.3%), Nigeria (5.0%), and the Netherlands (4.2%). The majority of the 3,194 articles were indexed in PubMed (90.0%). The top 10 journals (Table 3) represented 29.9% of the entries, and comprised journals with a wide range of impact factors (1.3-33.6). The top 10 countries among the 3,107 articles for which the country of the first author’s affiliating institute was known (97.3%) represented 64.2% of all entries (Table 4). Compared to the other countries in the list, Nigeria and India had the lowest percentage of articles indexed in PubMed, the lowest percentage of articles where the journal had an impact factor or article influence score, and the lowest median impact factor and article influence score (Table 4). Figure 4 shows the evolution of accessibility of articles through the internet, whereby limited access (by registration) peaked in 2007, to be overtaken by open internet access in 2010.

**Table 3:** Top 10 journals for 3,194 English-language articles in the Malaria in Pregnancy Library, January 2012

	**Journal**	**Number**	**% of total**	Impact factor*	**Eigenfactor score**†	**Article influence score** (**AIS**)†	**Start year journal**
1	***American Journal of Tropical Medicine and Hygiene***	184	5.8	2.446	0.03423	0.910	1952
2	***Malaria Journal***	158	4.9	3.489	0.01855	0.999	2002
3	***Transactions of the Royal Society of Tropical Medicine and Hygiene***	131	4.1	2.832	0.01495	0.811	1920
4	***Journal of Infectious Diseases***	105	3.3	6.288	0.11262	2.346	1904
5	***Tropical Medicine and International Health***	74	2.3	2.841	0.01791	0.995	1996
5	***Lancet***	74	2.3	33.633	0.37864	12.715	1823
7	***Annals of Tropical Medicine and Parasitology***	70	2.2	1.288	0.00458	0.534	1907
8	***Infection and Immunity***	58	1.8	4.098	0.09672	1.331	1970
9	***PLoS ONE***	53	1.7	4.411	0.31957	1.941	2006
10	***British Medical Journal***	47	1.5	13.471	0.14250	4.646	1857

*2010 JCR Science Edition, ISI Web of Knowledge, Thomson Reuters [8].

†2010 http://www.eigenfactor.org/index.php[9].

**Table 4:** Common countries of first-author affiliations for 3,107 English-language articles in the Malaria in Pregnancy Library, January 2012

	**Country**	**Number**	**Articles in active journals (%)**	**Articles indexed in PubMed (%)**	**Articles in journals with impact factor (%)**	**Median impact factor** (**range**)	**Articles in journals with AIS (%)**	**Median AIS(range)**
1	**USA**	669	639 (95.5)	627 (93.7)	571 (85.4)	3.55 (0.28-53.49)	565 (84.5)	1.23 (0.06-21.35)
2	**UK**	384	339 (88.3)	333 (86.7)	293 (76.3)	3.49 (0.44-36.10)	289 (75.3)	1.07 (0.17-19.31)
3	Nigeria*	269	246 (91.4)	174 (64.7)	111 (41.3)	1.25 (0.16-36.10)	81 (30.1)	0.53 (0.01-19.31)
4	**Thailand**	124	115 (92.7)	118 (95.2)	104 (83.9)	3.03 (0.44-53.49)	103 (83.1)	0.91 (0.17-21.35)
5	**India**†	123	116 (94.3)	96 (78.0)	60 (48.8)	1.59 (0.30-33.63)	47 (38.2)	0.58 (0.16-12.72)
6	**France**	120	118 (98.3)	119 (99.2)	115 (95.8)	3.49 (0.97-33.63)	115 (95.8)	0.99 (0.35-12.72)
7	**Australia**	113	107 (94.7)	103 (91.2)	100 (88.5)	4.10 (0.59-35.20)	94 (83.2)	1.33 (0.34-16.12)
8	**Tanzania**	82	71 (86.6)	71 (86.6)	43 (52.4)	2.79 (0.30-33.63)	42 (51.2)	0.91 (0.35-12.72)
9	**Kenya**	68	64 (94.1)	60 (88.2)	49 (72.1)	2.84 (1.08-33.63)	48 (70.6)	0.99 (0.35-16.82)
9	**Denmark**	68	65 (95.6)	66 (97.1)	66 (97.1)	4.10 (1.60-33.63)	64 (94.1)	1.33 (0.48-12.72)

Abbreviations: *AIS* Article Influence Score.

*Nigeria: comparing percentage of articles indexed in PubMed to any other country in the list: Chi-square test p < 0.05; comparing the presence of an impact factor and article influence score of a journal to any other country in the list: Chi-square test p < 0.05 except for India and Tanzania for the presence of an impact factor, and India for the presence of an article influence score; comparing the median impact factor and median article influence score for Nigeria to any other country in the list: (non-parametric test) p < 0.05 except for India for the median article influence score.

†India: comparing percentage of articles indexed in PubMed to any other country in the list: Chi-square test p < 0.05 except for Tanzania and Kenya; comparing the presence of an impact factor and an article influence score to any other country in the list: Chi-square test: p < 0.05 except for Nigeria and Tanzania; comparing the median impact factor and the median article influence score for India to any other country in the list: (non-parametric test) p < 0.05 except for Nigeria for the median article influence score.

## Articles with malaria in pregnancy as main theme

**Figure 5:**
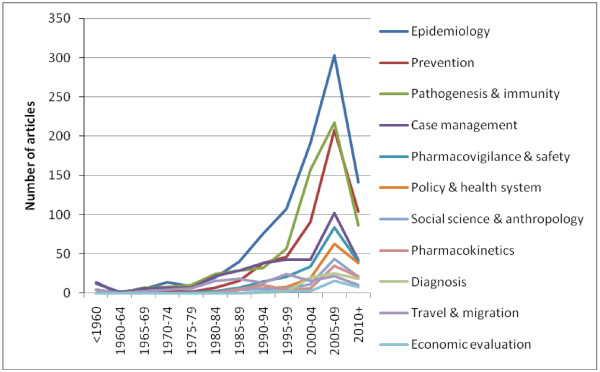
**Topics over time among 1,793 English-language articles with malaria in pregnancy as main theme, January 2012.** *Note that the X-axis indicates five-year time intervals and that for 2010+, only data for two out of the five years has been collected so far.

Of the 3,194 articles, 1,846 (57.8%) had MiP as the main theme and 1,793 (97.1%) were available in portable document format (PDF) for further evaluation by topic (Table 5, Figure 5). Most articles involved original research, and the USA, UK and Nigeria were the most common countries of the first author institute.

**Table 5:** Topics among 1,793 English journal articles with malaria in pregnancy as main theme, Malaria in Pregnancy Library, January 2012

Malaria in Pregnancy Topic*	**Total**	**Content**†			**Top 3 countries of institute of first author**		
	**1,793**	**Original research (%)**	**Review (%)**	**Other (%)**	**Country 1 (%)**	**Country 2 (%)**	**Country 3 (%)**
**Epidemiology and Burden**	920	761 (82.7)	125 (13.6)	91 (9.9)	Nigeria 144 (15.7)	USA 135 (14.7)	UK 95 (10.3)
**Pathogenesis and Immunity**	631	520 (82.4)	84 (13.3)	34 (5.4)	USA 157 (24.9)	France 57 (9.0)	UK 50 (7.9)
**Prevention**	520	380 (73.1)	112 (21.5)	100 (19.2)	USA 106 (20.4)	Nigeria 71 (13.7)	UK 53 (10.2)
**Case Management**	353	276 (78.2)	61 (17.3)	37 (10.5)	USA 55 (15.6)	Thailand 46 (13.0)	India 36 (10.2)
**Pharmaco**-**vigilance and Drug Safety**	209	151 (72.3)	46 (22.0)	22 (10.5)	USA 53 (25.4)	Thailand 38 (18.2)	UK 17 (8.1)
**Travel and Migration**	136	100 (73.5)	23 (16.9)	23 (16.9)	USA 44 (32.4)	UK 19 (14.0)	Australia 9 (6.6)
**Policy and Health Systems**	138	96 (69.6)	34 (24.6)	63 (45.7)	USA 26 (18.8)	UK 20 (14.5)	Nigeria 16 (11.6)
**Social Science and Anthropology**	91	84 (92.3)	7 (7.7)	25 (27.5)	Nigeria 22 (24.2)	Tanzania 11 (12.1)	UK 10 (11.0)
**Pharmaco**-**kinetics**	86	62 (72.1)	21 (24.4)	7 (8.1)	USA 21 (24.4)	Thailand 18 (20.9)	Australia/UK 7 (8.1)
**Diagnosis**	66	63 (95.5)	2 (3.0)	4 (6.1)	USA 13 (19.7)	Nigeria 8 (12.1)	Germany 8 (12.1)
**Economics**	32	25 (78.1)	8 (25.0)	12 (37.5)	UK 8 (25.0)	USA 7 (21.9)	Kenya 3 (9.4)

*One article can cover more than one topic.

† Categories under content are not mutual exclusive.

**Figure 6:**
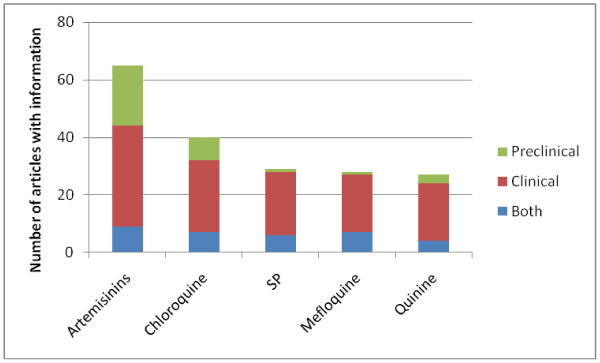
English-language articles with malaria in pregnancy as main theme and information on pharmacovigilance and safety by anti-malarial, January 2012.

The main topic category was “epidemiology and burden” (920 or 51.3%). “Pathogenesis and immunity” articles were the second most popular topic category (631 or 35.2%). Articles with chondroitin or chondroitin sulphate A (CSA), an important receptor for placental malaria, in the title (102) first appeared in 1996 - the year of the landmark article from Fried and Duffy, on adherence of *Plasmodium falciparum* to CSA in the placenta - with a peak in 2010 (14 articles) [14]. “Prevention” was the third most popular topic category (520, 29%), with articles on chemoprophylaxis (200 or 38.5%), intermittent preventive therapy in pregnancy (248 or 47.7%), nets or insecticide-treated nets (181 or 34.8%), indoor residual spraying (36 or 6.4%) and insect repellents (8 or 1.4%). Articles on chemoprophylaxis were among the earliest studies in this category (nine articles published prior to 1980) and the number of studies increased substantially in the late 1980s. Articles on IPTp and ITNs use in pregnancy started to emerge in the mid-1990s [15-17]. To date, six articles have been published with information on intermittent screening and treatment in pregnancy (ISTp) [18-23]. The “case management” category (353, 19.7%) included clinical case reports/series and drug-efficacy studies; the former were predominantly from institutes in the USA whereas Thailand ranked first among the countries conducting drug-efficacy studies. Artemisinin derivatives (40), chloroquine (35), quinine (35), SP (25), and mefloquine (21) were the most commonly evaluated drugs in studies. Most articles in the “pharmacovigilance and drug safety” category (209, 11.7%) were on clinical pharmacovigilance (169 or 80.9%), and 67 or 32.1% were on preclinical pharmacovigilance. Specific drugs or compounds were discussed in 190 articles (90.9%); anti-malarials in 153 articles and insecticides in 37 articles. There were relatively more reports on artemisinin derivatives (66), in particular preclinical studies (30), than the other anti-malarials commonly used in pregnancy (Figure 6). The “Pharmacokinetics” category (86, 4.8%) included articles on the pharmacokinetics of both antimalarials (75 or 87.2%) and insecticides (11 or 12.8%) in pregnancy. Among these, the main antimalarials evaluated in articles reporting on specific drugs were the same as for pharmacovigilance: Chloroquine (22), artemisinin derivatives (20), SP (12), mefloquine (7) and quinine (7). From the 1980s onwards, there has been a steady output of articles related to travel and migration and MiP, averaging about five per year. Articles on policy and health systems research (138, 7.7%), and social science and anthropology (91, 5.1%) first appeared in the late 1980s, with a significant increase since 2000. There were few articles published on the economics of malaria in pregnancy (32, 1.8%), again first appearing in the 2000s. The number of articles in the “diagnosis” category (66, 3.7%) has seen a rise since the late 1990s. The top 15 articles by citation can be seen in Table 6; five were review articles and 10 comprised original research. Five involved important immunological discoveries and two articles were trials on intermittent preventive treatment which assisted in providing the evidence to use this strategy for prevention.

**Table 6:** Top 15 English-language articles with malaria in pregnancy as main theme by citations, May 2012*

	**Authors**	**Title**	**Citation**	**Citations**/**year**	**Type of article**
1	Fried et al. 1996 [14]	Adherence of *Plasmodium falciparum* to chondroitin sulphate A in the human placenta	680	40.0	Original research
2	Brabin 1983 [24]	An analysis of malaria in pregnancy in Africa	588	19.6	Review
3	Steketee et al. 2001 [5]	The burden of malaria in pregnancy in malaria-endemic areas	527	43.9	Review
4	McGregor 1984 [25]	Epidemiology, malaria and pregnancy	398	13.7	Review
5	Fried et al. 1998 [26]	Maternal antibodies block malaria	391	26.1	Original research
6	McGregor et al. 1983 [27]	Malaria infection of the placenta in The Gambia, West Africa: its incidence and relationship to stillbirth, birth weight and placental weight	326	13.7	Original research
7	Parise et al. 1998 [28]	Efficacy of sulphadoxine-pyrimethamine for prevention of placental malaria in an area of Kenya with a high prevalence of malaria and human immunodeficiency virus	312	20.8	Original research
8	Salanti et al. 2003 [29]	Selective upregulation of a single distinctly structured var gene in chondroitin sulphate A-adhering *P*. *falciparum* involved in pregnancy-associated malaria	308	30.8	Original research
9	Shulman et al. 1999 [30]	Intermittent sulphadoxine-pyrimethamine to prevent severe anaemia secondary to malaria in pregnancy: a randomized placebo-controlled trial	299	21.4	Original research
10	Beeson et al. 2000 [31]	Adhesion of *P*. *falciparum*-infected erythrocytes to hyaluronic acid in placenta	259	19.9	Original research
11	Menendez 1995 [32]	Malaria during pregnancy: a priority area of malaria research and control	254	14.1	Review
12	Desai et al. 2007 [4]	Epidemiology and burden of malaria in pregnancy	244	40.7	Review
13	Steketee et al. 1996 [33]	The effect of malaria and malaria prevention in pregnancy on offspring birth weight, prematurity and intrauterine growth retardation in rural Malawi	244	14.4	Original research
14	Menendez et al. 2000 [34]	The impact of placental malaria on gestational age and birth weight	241	18.5	Original research
15	Fried et al. 1998 [35]	Malaria elicits type 1 cytokines in the human placenta; IFN-λ and TNF-α associated with pregnancy outcomes	233	15.5	Original research

*Obtained using Google scholar and “Publish or Perish” [12,13].

## Reports

By January 2012, there were 619 English-language reports (13.3% of the library entries); the earliest report was from 1950. Again, there was an exponential increase in reports from 2000 onwards, with a total of 31 reports between 1950 and 2000 and 503 reports between 2001 and 2010, with 2006 and 2008 as the most productive years (81 and 80 reports published respectively). Only 74 reports (12.0%) had MiP as main theme. Original research was reported by 16.0%, and 22.1% were reviews. A large proportion of reports were policies (49.6%) or programme evaluations (31.8%), and some were practice guidelines (6.3%). Examples of reports include national strategic plans for malaria control, Global Fund Proposals (the Global Fund to Fight AIDS, Tuberculosis and Malaria) with information on strategies to control MiP, Demographic and Health Surveys with a section on MiP prevention, World Health Organization (WHO) or Roll Back Malaria (RBM) reports with guidelines or indicators of progress on MiP, and reports from governmental aid organizations with information on MiP such as from the United States Agency for International Development (USAID) and The President’s Malaria Initiative (PMI) or from other technical agencies such as JHPIEGO, an international non-profit health organization affiliated with Johns Hopkins University. The top two countries where the corporate institutes were based were the USA, with 187 reports (mainly USAID and PMI), and Switzerland with 82 reports (mainly WHO and RBM based in Geneva). There were 19 reports for Kenyan institutes and 18 for South African institutes. The country of primary author institute could not be identified for 36 reports.

## Theses

There were 173 English-language theses in the Library (3.7% of library entries); all were produced between 1972 and 2011, 67.1% of which in the last 10 years. Of these, 128 (74.0%) had MiP as main theme, and 86 were PhD theses (65.7%). Only 23 were freely available on the internet (13.3%), 60 (34.7%) had password-protected access, and 90 were not available on the internet.

## Registered trials and time to publication

Sixty four studies with MiP key words in the title were identified of which 49 (76.6%) were randomized trials. The majority of studies were registered in clinicaltrials.gov (56) [36], although there was significant overlap between registers with some studies registering with more than one registry. The study status for these 64 studies was as follows: seven were ‘suspended’ or ‘terminated’ (five due to lack of sufficient malaria in the study area to be able to achieve the study objectives with the available funds, one because new information that the study drug was not suitable for use for pregnancy became available, and for one study the reason was not provided); four were not yet recruiting; 17 were recruiting; 33 were completed and for three the status was unclear. For 23 completed studies (69.7%), a publication with associated study results could be identified (one thesis and 22 articles). The mean interval between the end of the study and publication was 1.9 years (SD 1.2, range 0–4 years). For the 10 completed studies without a publication, the mean interval between the study completion date and the review date (December 2011) was 2.6 years (SD 1.6, range 0–5 years). Seven of these studies were clinical trials, and five of them were completed more than two years ago.

## Conference proceedings and time to publication

The proceedings of three MIM conferences held in 2002, 2005 and 2009 were evaluated. In 2002, 4.8% (28/578) of the abstracts had MiP key words in the title, in 2005 this was 8.5% (60/708) and in 2009 this was 7.7% (65/849). Of the 153 abstracts, the vast majority involved studies in Africa (83.0%), with Nigeria as the country contributing the most (13.1% or 20); 9.8% were categorized as originating from developed countries (mainly laboratory-based studies), two were from Asia (1.3%), and for 5.9% it was not clear. A formal publication from the studies described in the original abstract could be retrieved for 56.2% of the abstracts (67.9% in 2002, 61.7% in 2005 and 46.2% in 2009). Twenty-seven publications (32.1%) linked to the abstracts were in the public domain before the conference, and for the remaining 59 abstracts, the median time between conference and publication date was 16.2 months (range two to 81 months after the conference). In 73.3%, the first author on the publication was the same as in the original abstract. Abstracts from studies in Africa were less likely to result in a formal publication (68/127 or 53.5%) than abstracts from other continents (15/17 or 88.2%, p < 0.05). Fourteen abstracts reported trials, and for 10 of them a publication was retrieved (71.4%). Of the four abstracts involving trials for which no formal publication could be retrieved, one of them was in a study register. For the 10 trials with a positive finding (defined as a significant difference between study arms as far as available information allowed to assess), a formal publication could be retrieved for seven (70.0%); for the four trials with a negative finding (no significant differences between study arms), three formal publications were retrieved (75.0%, p = 1.00).

## Malaria research in children *vs* pregnant women

**Figure 7:**
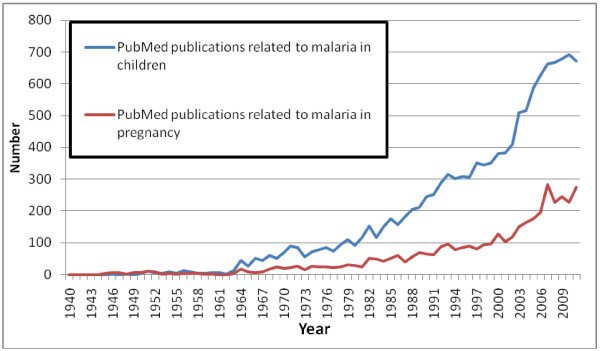
PubMed publications on malaria related to children or pregnancy for 1940–2011.

A search in PubMed using the terms for child and malaria as described in Methods generated 12,352 references, compared to 3,928 references generated from a search for pregnancy and malaria using the terms in Table 1 (conducted on 20 March 2012, Figure 7). For both vulnerable groups, the number of articles started to increase significantly at about the same time (1960s), reaching a plateau around 2007; the average annual ratio of number of articles on children to number of articles on pregnancy between 1970 and 1999 was 3.5 and between 2000–2011 was 3.1 (*t*-test, p = 0.008) (Figure 7).

## Discussion

This analysis illustrates the considerable growth in the literature available on MiP over the years, most notable in the past decade (2000–2011) when the overall number of publications on MiP has more than doubled. The most probable explanation for the rapid increase in literature on MiP is the increased interest in MiP research, which was stimulated through political interest in and prioritization for malaria control through a series of conferences from Amsterdam (1992), Harare (1997) and Abuja (2000) [3]. This also contributed to growth in the availability of funding over the last decade, some of which is provided by new funding organizations. Most notable are: the Bill and Melinda Gates Foundation, which started to disburse research grants to institutions for malaria research in 1999; the European and Developing Countries Clinical Trials Partnership (EDCTP), established in 2003 to accelerate the development of new or improved drugs, vaccine and diagnostics against HIV/AIDS, malaria and tuberculosis; the National Institutes of Health (NIH); the Wellcome Trust; and the European Union. The growing interest in MiP research culminated in 2007 with the establishment of the MiP Consortium, a global network of 41 partners joined together to improve the control of malaria in pregnancy in Africa, Asia and Latin America [37].

Pregnant women receive relatively less attention than children; for every publication on malaria in pregnancy, there are about three publications on malaria in children, but this ratio seemed to have decreased marginally over time. The exponential increase in available publications, such as those illustrated in Figures 3 and 7 are not unique for malaria and have been reported for a range of health topics [38], suggesting that this may have been facilitated by improved economic conditions and availability of research funds.

The internet as a source and archive for documentation may in itself be a reason for the growth in research and hence literature seen over the last two decades. Many journals started indexing articles in PubMed from around 1965, and an approximate 40% increase in PubMed publications has been reported in the period 1995–2004 [39]. The availability of internet access globally has facilitated the retrieval of information which otherwise would have been difficult to access (Figure 4). The increased use and accessibility of the internet has resulted in changes in publishing practice, with an increase in the number of open-access journals and journals that publish exclusively in electronic format. The prominence of the contributions of the entirely open-access journals *Malaria Journal* and *PLoS ONE* to the MiP literature is remarkable given the youth of these journals, which started publishing in 2002 and 2006, respectively; they contributed 4.9% and 1.7% respectively of all English-language articles, *vs* e.g. 4.1% for the *Transactions of the Royal Society of Tropical Medicine and Hygiene* which started publishing in 1920, or 3.3% for the *Journal of Infectious Diseases* which started publishing in 1904.

In the analysis of articles with MiP as the main theme, epidemiology and burden was the most common topic, whereas social science and anthropology, pharmacokinetics, diagnosis and economics were the least common. The number of articles in the category “diagnosis” has seen a rise since the late 1990s with the expansion of diagnostic options, such as rapid diagnostic tests for malaria and increased use of polymerase chain reaction technology. The number of economic evaluations to date is low. However, it is anticipated that the number of economic studies will grow more substantially because cost-effectiveness analyses are now routinely conducted alongside large-scale clinical trials evaluating new interventions. Furthermore, the cost effectiveness of current interventions is likely to change with decreasing malaria transmission as a result of the renewed malaria control and elimination efforts. Interest in the pharmacokinetics of anti-malarials in pregnancy has arisen with the realization that pregnancy may affect drug metabolism, and dose adjustments for some drugs are needed in order for these drugs to be as effective as in non-pregnant adults [40]. It is therefore surprising that the first pharmacokinetic study of SP among pregnant women was published only about 10 years *after* the drug was first used for intermittent preventive treatment [41]. Drugs used in pregnancy should be safe for both mother and the unborn child, and newer drugs, such as the artemisinin derivatives, are now more rigorously screened for safety and have received relatively more attention (particularly in preclinical safety studies) than any other drug. The availability of newer techniques to assess drug safety in the last decade [42], and concerns about the embryotoxic effects of the artemisinin class of anti-malarials at low dose ranges in animal models [43], may also have contributed to this increased attention.

Several measures are used to evaluate journal quality and influence. The impact factor is a measure reflecting the average number of citations to recent articles published in journals whereas the Eigenfactor score uses an iterative weighting system to calculate a summary index that reflects both the quality and the quantity of citations [10]. The article influence score (AIS) divides the Eigenfactor score by the number of articles in the journal; to facilitate interpretation, the AIS is normalized, so that the mean article in the *Journal of Citation Reports* has an AIS of 1.00 [10,44]. Both EigenFactor and AIS use publications over the previous five years, whereas impact factor uses publications over the previous three years [10]. However, all these different measures have shown a high correlation [10,44]. In the context of this analysis, Tables 3 and 4 show substantial variation in quality between journals contained in the MiP Library, and in the average quality (as quantified by the median impact factor) of the journals by country of the first-author affiliation. Although Nigeria is frequently mentioned in the top three countries of institute of first author (Tables 4 and 5), the journals used are less likely to be indexed in PubMed or to have an impact factor, and for those journals which do, the median is significantly lower than for the other countries reported. This is also the case for India although to a lesser extent. The under-representation of African journals in PubMed and the lack of quality is a known issue, and efforts have been documented to improve this imbalance [45].

By comparing publications against entries in trial registers, it was possible to identify studies which were conducted but for which no formal publication was available. About 33% of MiP trials, which were completed according to study registers, had not yet been published within two years. This may not be specific for malaria in pregnancy. A similar analysis among trials (all diseases) found that 32% was not published, whereas the time to publication was very similar [46]. The sub-analysis of abstracts generated by the MIM conferences showed that up to 36% of research or programme evaluations on MiP may not make it into a formal publication within five years. This included three trials which had not been registered. Trial registration is likely to increase in the future because of the requirements by funding agencies and major journals for trial registration.

Limitations of the MiP Library include a likely bias towards material which is (freely) available through the internet as opposed to very old articles, theses, books, book chapters or internal reports which are not available electronically and are more difficult to obtain. Another limitation is the inclusion of all material regardless of source or quality, such that the Library contains both high and low quality articles and other materials. Although information on MiP in all languages is included that are identified, the analysis was restricted to English language articles given the incompleteness of articles in other languages. This study has potential limitations in selection bias and misclassification, e.g. in the analysis of sub-topics, only publications were used for which a PDF was available. Although the country of the first-author affiliation was used as an indicator of the country of the publication, it is recognized that articles are generally a group effort and can represent authors from many institutes in many countries.

## Conclusions

The last decade has seen a dramatic increase in the number of publications related to malaria in pregnancy, indicating a significant positive trend in research interest and funding. An increasing proportion of these publications are publically available through online sources. The MiP Library is a unique online repository, capturing articles indexed in PubMed and other online sources of peer-reviewed research articles, as well as non-indexed articles, reports and other material with information on MiP, and is an excellent scholarly source for literature and systematic reviews.

## Abbreviations

ANC: Antenatal clinic; CSA: Chondroitin sulphate A; ITN: Insecticide treated nets; IPTp: Intermittent preventive treatment in pregnancy; MiP: Malaria in pregnancy; PMI: President’s Malaria Initiative; RBM: Roll Back Malaria Partnership; SP: Sulfadoxine-pyrimethamine; WHO: World Health Organization.

## Competing interests

The authors declare that they have no competing interests.

## Authors’ contributions

FOtK and JH conceived the concept of the project. SP, AR, and HW compiled the MiP Library database and AMvE, JH, AR and HW maintain it. AMvE conducted the analysis and wrote the first draft of the manuscript and JH and FOtK revised it. All other authors reviewed the final version.

## Supplementary Material

Additional file 1 Current sources for the Malaria in Pregnancy Library. Click here for file
