# Supplementary material for: The Malaria in Pregnancy Library: a bibliometric review
Source: Malar J. 2012 Oct 30;11:362. doi: 10.1186/1475-2875-11-362 (PMC3522037; doi:10.1186/1475-2875-11-362)
Supplement: Additional file 1 — Current sources for the Malaria in Pregnancy Library. [file 1475-2875-11-362-S1.pdf]

Annex 1 Current sources for the Malaria in Pregnancy library in alphabetical order (websites checked on 12 February 2012)

Name database	Description	Web site
**AJOL**	African Journals OnLine is the world's largest and pre-eminent collection of peer-reviewed, African-published scholarly journals. This is a non-profit organization based in South Africa	http://www.ajol.info/
**Bioline**	Bioline International provides open access to peer reviewed bioscience journals published in developing countries.	http://www.bioline.org.br/journals
**CINAHL**	Cumulative Index to Nursing and Allied Health Literature	http://www.ebscohost.com/cinahl/
**CSA Illumina**	Cambridge Scientific Abstracts. The interface provides access to more than 100 databases published by CSA and its partners.	http://www.csa.com/csaillumina/login.php
**Directory of open access journals (DOAJ)**	Free, full text, quality controlled scientific and scholarly journals, covering all subjects and many languages	http://www.doaj.org/
**Eldis**	A database for global development information on international development issues	http://www.eldis.org/
**Emerald**	Emerald Group Publishing has a strong presence in disciplines such as social sciences	http://www.emeraldinsight.com/
**FDA**	The food and drug administration is an agency within the US Department of Health and Human Services. The FDA is protecting the public health by assuring the safety, effectiveness and security of human drugs and vaccines	http://www.fda.gov/
**Global Health Library**	The objective of the Global Health Library (GHL), launched in 2005, is to contribute to radically increase access to information and scientific evidence on health, particularly in developing regions. GHL is promoted and led by WHO as part of its strategy of knowledge management in global public health. The GHL aims to 'strengthen, promote and develop worldwide networks on the collection, organization, dissemination and universal access to reliable health sciences	http://www.globalhealthlibrary.net/php/index.php

	information'.	
**Google**	General search engine	http://www.google.com/
**Google Scolar**	Search engine for scholarly papers	http://scholar.google.com/
**International Health Links Centre**	The goal of the IHLC is to enhance access to health care in the developing world by promoting international partnerships that will increase the number and skills of the health workforce	http://www.ihlc.org.uk/
**LILACS (Spanish/ Portuguese)**	Coordinated by BIREME, LILACS has completed 25 years of cooperation for the strengthening of the flows of technical and scientific health information in Latin America and the Caribbean	http://bases.bireme.br/cgi-bin/wxislind.exe/iah/online/?IsisScript=iah/iah.xis&base=LILACS&lang=i&form=F
**Medecine d'Afrique Noire**	First international pan-african medical journal	http://www.santetropicale.com/club/manelec/index.asp
**NLM Catalog**	The NLM Catalog provides access to NLM bibliographic data for journals, books, audiovisuals, computer software, electronic resources and other materials	http://www.ncbi.nlm.nih.gov/nlmcatalog
**Popline**	POPLINE® (POPulation information onLINE) contains citations with abstracts to scientific articles, reports, books, and unpublished reports in the field of population, family planning, and related health issues.	http://www.popline.org/
**Proquest**	Search engine to the world's knowledge – from dissertations to governmental and cultural archives to news, in all its forms. Particularly useful for theses.	http://www.proquest.co.uk/en-UK/
**PubMed**	PubMed comprises more than 21 million citations for biomedical literature from MEDLINE, life science journals, and online books.	http://www.ncbi.nlm.nih.gov/pubmed/
**Quertle**	Quertle goes beyond simple term matching to identify the most salient information in the literature. Using a combination of linguistic methods, Quertle finds facts defined within documents, creating its own database of **about 250 million relationships**, and is able to report the ones that are relevant to your query. Quertle's approach is based on a thorough understanding of biology and chemistry and was built from the	http://www.quertle.info/

	ground up to address the unique needs of this technical literature.	
**Scirus**	Scirus is a comprehensive scientific research tool on the web.	http://www.scirus.com/
**Scopus**	Scopus is an abstract and citation database of research literature and quality web sources	http://www.scopus.com/home.url
**Web of Knowledge**	Citation and journal database; this database screens Web of Science, BIOSIS Citation Index, BIOSIS Previews, MEDLINE and Journal Citation Reports, and scientific web sites.	http://wok.mimas.ac.uk/
**WHO library**	World Health Organization Library and Information networks for knowledge database (WHOLIS)	http://dosei.who.int/
**WorldCat**	WorldCat is the world's largest network of library content and services. Includes other databases like OAIster.	http://www.worldcat.org/
**For theses:**		
**Index to theses**	A comprehensive listing of theses with abstracts accepted for higher degrees by universities in the United Kingdom and Ireland since 1716.	http://www.theses.com/
**Networked Digital Library of Theses and Dissertations**	The Networked Digital Library of Theses and Dissertations (NDLTD) is an international organization dedicated to promoting the adoption, creation, use, dissemination, and preservation of electronic theses and dissertations (ETDs).	http://www.ndltd.org/
**Proquest Dissertations and Theses**	ProQuest Dissertations and Theses — Full text is the world's most comprehensive collection of dissertations and theses. The official digital dissertations archive for the Library of Congress and the database of record for graduate research. PQDT — Full Text includes 2.7 million searchable citations to dissertation and theses from around the world from 1861 to the present day together with 1.2 million full text dissertations that are available for download in PDF format. Over 2.1 million titles are available for purchase as printed copies. The database offers full text for most of the dissertations added since 1997 and strong retrospective full text coverage for older graduate	http://www.proquest.com/en-US/catalogs/databases/detail/pqdt.shtml

	works.	
**For registered studies**		
**ClinicalTrials.gov**	Registry and results database of federally and privately supported clinical trials conducted in the United States and round the world	http://clinicaltrials.gov/
**RePorter**	National Institute of Health. Research Portfolio Online Reporting Tools. Reports, data and analyses of NIH research activities (formerly known as CRISP).	http://projectreporter.nih.gov/reporter.cfm
**Current controlled trials**	Current Controlled Trials allows users to search, register and share information about randomised controlled trials. This database allows searches across multiple registers	http://www.controlled-trials.com/
**Pan African Clinical Trials Registry**	This is a regional register of clinical trials conducted in Africa.	http://www.pactr.org/
**International Clinical Trials Registry Platform Search Portal**	This database provides access to a central database containing the trial registration data sets provided by the following registries: Australian New Zealand Clinical Trials Registry; ClinicalTrials.gov; and ISRCTN.	http://apps.who.int/trialsearch/
**Institutional websites**		
**Global Fund**	The Global Fund to Fight AIDS, Tuberculosis and Malaria is an international financing institution that invests the world's money to save lives.	http://www.theglobalfund.org/en/
**Jhpiego**	Jhpiego is an international non-profit health organization affiliated with The Johns Hopkins University. For more than 35 years and in over 150 countries, Jhpiego has worked to prevent the needless deaths of women and their families.	http://www.jhpiego.org/
**Malaria Indicator Surveys**	In these nationally representative household surveys, key malaria indicators are measured.	http://www.malariasurveys.org/
**Measure DHS**	The Demographic and Health Surveys program has collected, analyzed and disseminated representative data on population health, HIV and nutrition through more than 200 surveys in over 75 countries.	http://www.measuredhs.com

**PMI**	The President's Malaria Initiative (PMI) is a five-year plan of U.S. Government resources to reduce the burden of malaria on the African continent.	http://www.pmi.gov/
**PSI-malaria**	Population Services International provides malaria control support to national Ministries of Health in over 30 countries worldwide.	http://www.psi.org/our-work/healthy-lives/malaria
**WHO malaria**	The World Health Organization Global Malaria Programme (GMP) convenes experts to review evidence and set global policies. GMP's policy advice provides the benchmark for national malaria programmes and multilateral funding agencies.	http://www.who.int/malaria/about_us/en/index.html<br>http://www.who.int/topics/malaria/en/
**Roll Back Malaria Partnership**	The RBM Partnership is the global framework for coordinated action against malaria	http://www.rbm.who.int/
